# Identification and experimental validation of mitochondria pathway related genes in acute myeloid leukemia

**DOI:** 10.3389/fimmu.2026.1765805

**Published:** 2026-05-19

**Authors:** Rui Dou, Wei Li, Lei Zhang, Dan Li, Wei Cheng, Zunmin Zhu

**Affiliations:** 1 Institute of Hematology, Henan Key Laboratory of Stem Cell Differentiation and Modification, Henan Provincial People’s Hospital, People’s Hospital of Zhengzhou University, Zhengzhou, China; 2 Department of Hematology, Henan Provincial People’s Hospital, Zhengzhou University People’s Hospital, Henan University People’s Hospital, Zhengzhou, China

**Keywords:** acute myeloid leukemia, biomarker, mitochondria, prognosis, survival

## Abstract

**Background:**

Acute myeloid leukemia (AML) is a hematologic malignancy characterized by heterogeneity, poor prognosis, and limited biomarkers for risk prediction. Mitochondria pathway related genes (MPRGs), as central regulators of cellular metabolism and immune microenvironment dynamics, may provide useful information for prognostic assessment and biological characterization in AML.

**Methods:**

MPRGs were obtained from the MitoCarta3.0 database. Univariate Cox and Kaplan-Meier methods were conducted to analyze their prognostic relevance. LASSO penalized regression followed by stepwise multivariate Cox analysis yielded an optimal gene panel in the TCGA-LAML dataset. External validation was performed across three GEO datasets (GSE10358, GSE106291, GSE71014). Finally, the role of UCP2 was examined *in vitro* by assessing UCP2 knockdown effects on MOLM-13 cell behavior.

**Results:**

A prognostic signature comprising seven MPRGs (UCP2, FAM162A, ACCS, HSD1, ACSF2, PPIF, and SDHA) was established. High-risk patients exhibited significantly shorter survival. The MPRGs risk score served as an independent predictor of prognosis. Moreover, elevated risk scores correlated with heightened immune checkpoint molecule expression and an immunosuppressive tumor microenvironment. UCP2 knockdown attenuated both proliferative capacity and migratory potential in MOLM-13 AML cells.

**Conclusion:**

In summary, the MPRG-based signature provides independent prognostic value in AML and reflects its association with an immunosuppressive microenvironment. These findings provide additional evidence that mitochondrial pathway-related genes are associated with AML prognosis and immune microenvironment features. UCP2 may represent a biologically relevant candidate gene in AML, although further mechanistic and clinical validation is required.

## 1 Introduction

Acute myeloid leukemia (AML) is an aggressive malignant clonal disease with poor survival arising from the myeloid progenitor cells, typified by uncontrolled blast expansion, hematopoietic function failure, and extramedullary involvement (1). Among adult acute leukemias, AML predominates with approximately 4.3 new diagnoses per 100,000 individuals annually, displaying remarkable heterogeneity (2, 3). AML treatment faces dual challenges of refractory disease and chemotherapy resistance, leading to induction failure rates of 40%-50% (4). Although therapeutic paradigms have evolved, including optimization of conventional chemotherapy, application of allogeneic transplantation, and emerging immunological interventions. Nevertheless, long-term outcomes remain poor: the five-year survival is about 40% for younger adults and only 5%-15% for those above 60 (5). Thus, clarifying disease biology, improving prognostic risk stratification, and identifying biologically relevant therapeutic candidates remain important challenges for AML management.

Mitochondria function as vital cellular organelles, serve as the principal bioenergetic hubs via oxidative phosphorylation (OXPHOS). They also play key roles in regulating programmed cell death, calcium flux, and reactive oxygen species (ROS) homeostasis (6, 7). Accumulating evidence implicates mitochondrial dysfunction in the pathogenesis and progression of hematological malignancies (8, 9). In AML, leukemic cells display pronounced metabolic reprogramming, which includes increased mitochondrial mass, elevated OXPHOS activity, and altered mitochondrial dynamics relative to normal hematopoietic cells (10, 11). This mitochondrial dependence represents a potential therapeutic vulnerability.

Beyond their metabolic functions, mitochondria are increasingly recognized as crucial regulators within the tumor immune microenvironment (TIME). Mitochondrial metabolism governs the differentiation, activation, and effector functions of various immune cell populations (12, 13). Dysfunctional mitochondria within tumor cells can release damage-associated molecular patterns (DAMPs), thereby provoking sterile inflammation and contributing to the formation of an immunosuppressive microenvironment (14). Furthermore, mitochondrial pathways modulate immune checkpoint protein expression and influence cancer cell susceptibility to immune-mediated killing (15, 16). Given the significant interplay between mitochondrial biology and immune regulation, mitochondria pathway-related genes (MPRGs) hold promise as prognostic biomarkers and therapeutic guides in AML management (17).

Herein, we systematically screened MPRGs with prognostic relevance in AML based on the MitoCarta3.0 database and TCGA-LAML cohort. A seven gene prognostic risk model was developed and subsequently validated across multiple independent datasets. We further investigated the correlation between the MPRG-derived risk score and characteristics of the tumor immune microenvironment. Finally, experimental validation was conducted to examine the functional role of UCP2 in AML cells.

## 2 Materials and methods

### 2.1 Data acquisition and processing

Transcriptomic profiles and corresponding clinical information of AML patients were extracted from The Cancer Genome Atlas (TCGA) database via the TCGAplot R package (version 8.0.0) (18). The TCGA-LAML served as the discovery cohort. Three Gene Expression Omnibus (GEO, https://www.ncbi.nlm.nih.gov/geo/) datasets, including GSE10358, GSE106291, and GSE71014, were employed for external validation. Somatic mutation data of TCGA-LAML patients were obtained from the Genomic Data Commons (GDC, https://gdc.cancer.gov/about-data/publications/pancanatlas). Computational analyses were executed in R (v4.5.0). The baseline characteristics of patients in the discovery and validation cohorts are summarized in **Supplementary Table 1**.

### 2.2 Identification of mitochondria pathway genes

The comprehensive list of mitochondria pathway genes (MPRGs) was downloaded from MitoCarta3.0 (https://www.broadinstitute.org/mitocarta).

### 2.3 Screening of prognostic MPRGs

Prognostically relevant MPRGs were filtered through two complementary approaches: univariate Cox proportional hazards modeling and Kaplan-Meier log-rank testing, implemented via tinyarray (v2.4.3). Genes meeting the significance threshold (*P* < 0.01) in both analyses were considered prognostically relevant MPRGs.

### 2.4 Construction of the MPRGs prognostic signature

Variable selection employed LASSO-penalized Cox regression via glmnet (v4.1-10), followed by stepwise multivariate Cox modeling using My.stepwise (v0.1.0) to finalize the gene panel. Individual risk scores were calculated as: Risk score = Σ (β_i × Expr_i), where β_i denotes the Cox coefficient and Expr_i represents normalized transcript abundance. The cohort was dichotomized at the median risk score threshold into high- and low-risk strata. Within each cohort, patients were divided into high-risk and low-risk groups using the median risk score as the cutoff value.

### 2.5 Validation of the prognostic model

The predictive performance of the MPRG signature was evaluated across discovery and validation datasets. Kaplan-Meier survival curves compared survival outcomes between risk categories. Time-dependent ROC curves were constructed using the time ROC R package (version 0.4) to evaluate the prognostic accuracy at 1-, 3-, and 5-year time points. The area under the curve (AUC) values were calculated accordingly. To determine whether the risk score serves as an independent prognostic factor, univariate Cox regression analysis was performed incorporating clinical variables including age and gender. A prognostic nomogram integrating significant variables was constructed using the rms R package (version 8.0-0). Model calibration was assessed by calibration curves, while decision curve analysis (DCA) via ggDCA (v1.2) evaluated net clinical benefit.

### 2.6 Differential expression and functional enrichment analyses

Differentially expressed genes (DEGs) between risk strata were identified via limma R package (version 3.66.2) with thresholds of |log_2_FC| > 1 and *P* < 0.05. Pathway annotation utilized clusterProfiler (v4.18.2) for KEGG and GO term enrichment. Hallmark pathway activity was interrogated through gene set enrichment analysis (GSEA) against MSigDB.

### 2.7 Mutational landscape analysis

Genomic alterations were characterized using maftools (v2.26.0). Oncoplot visualizations were generated to display the mutational landscape of each risk group.

### 2.8 Tumor microenvironment and immune infiltration analysis

Immune cell abundance was computationally inferred from previously established immune cell gene signatures using the ssGSEA algorithm implemented in the GSVA package (19). These scores should be interpreted as transcriptome-based estimates of immune infiltration rather than direct measurements of immune cell proportions. Pearson coefficients quantified associations between risk scores and checkpoint molecule expression. Additional immune-related signatures including MDSCs infiltration, T-cell dysfunction, and immune checkpoint blockade (ICB) resistance were evaluated using IOBR (version 0.99.0) (20).

### 2.9 Cell culture and quantitative real-time PCR

MOLM-13 human AML cells (ATCC) were propagated in RPMI-1640 containing 10% FBS and 1% antibiotics at 37 °C with 5% CO2. Lentiviral shRNA particles targeting UCP2 (LV-shUCP2) or scrambled controls (LV-shNC) were sourced from GENCEFE (Nanjing, China). Cells (2×10^5^) were infected with 25 µL Lentivirus (1.6×10^8^ TU/mL). RNA isolation utilized TRIzol (Invitrogen), with reverse transcription performed using PrimeScript RT Kit (Takara). Transcript quantification employed SYBR Green. Expression fold changes were derived via the 2^(-ΔΔCt)^ algorithm with β-actin normalization. UCP2 forward primer: CGGTTACAGATCCAAGGAGA, UCP2 reverse primer: GCGGACAGAGGCAAAGC. β-actin forward primer: TCAGGGTGAGGATGCCTCTC, β-actin reverse primer: CTCGTCGTCGACAACGGCT.

### 2.10 Transwell migration assay

Cell migration was evaluated using 24-well Transwell chambers with 8-μm pore size inserts (Corning). Cells (5 × 10^4^) suspended in serum-free medium were seeded in the upper chamber, while medium containing 20% FBS was added to the lower chamber as a chemoattractant. Following 24-hour incubation, transmigrated cells were fixed (4% paraformaldehyde), crystal violet-stained, and enumerated under a microscope.

### 2.11 Cell proliferation assay

Growth kinetics were monitored via CCK-8 colorimetric assay (Dojindo). Cells plated at 2×10³/well in 96-well format received 10 μL CCK-8 reagent at specified intervals (0, 24, 48, 72, 96 h). After 2-hour incubation, optical density at 450 nm was recorded.

### 2.12 Clinical sample collection and validation

Bone marrow aspirates from treatment-naïve AML cases (n = 10) and non-AML controls (n = 10) were procured at the Henan Provincial People’s Hospital under institutional ethics approval. All participants provided written consent. UCP2 expression was quantified by qRT-PCR as described above.

### 2.13 Statistical analysis

Statistical computations utilized R (v4.5.0). Group comparisons employed Student’s t-test or Wilcoxon rank-sum test based on data distribution. Survival differences were assessed via log-rank testing. Correlation analysis was assessed using Pearson’s rank correlation coefficient. Experiments included three biological replicates. Two-sided *P* < 0.05 denoted significance.

## 3 Results

### 3.1 Identification of MPRG in AML

**Figure 1:**
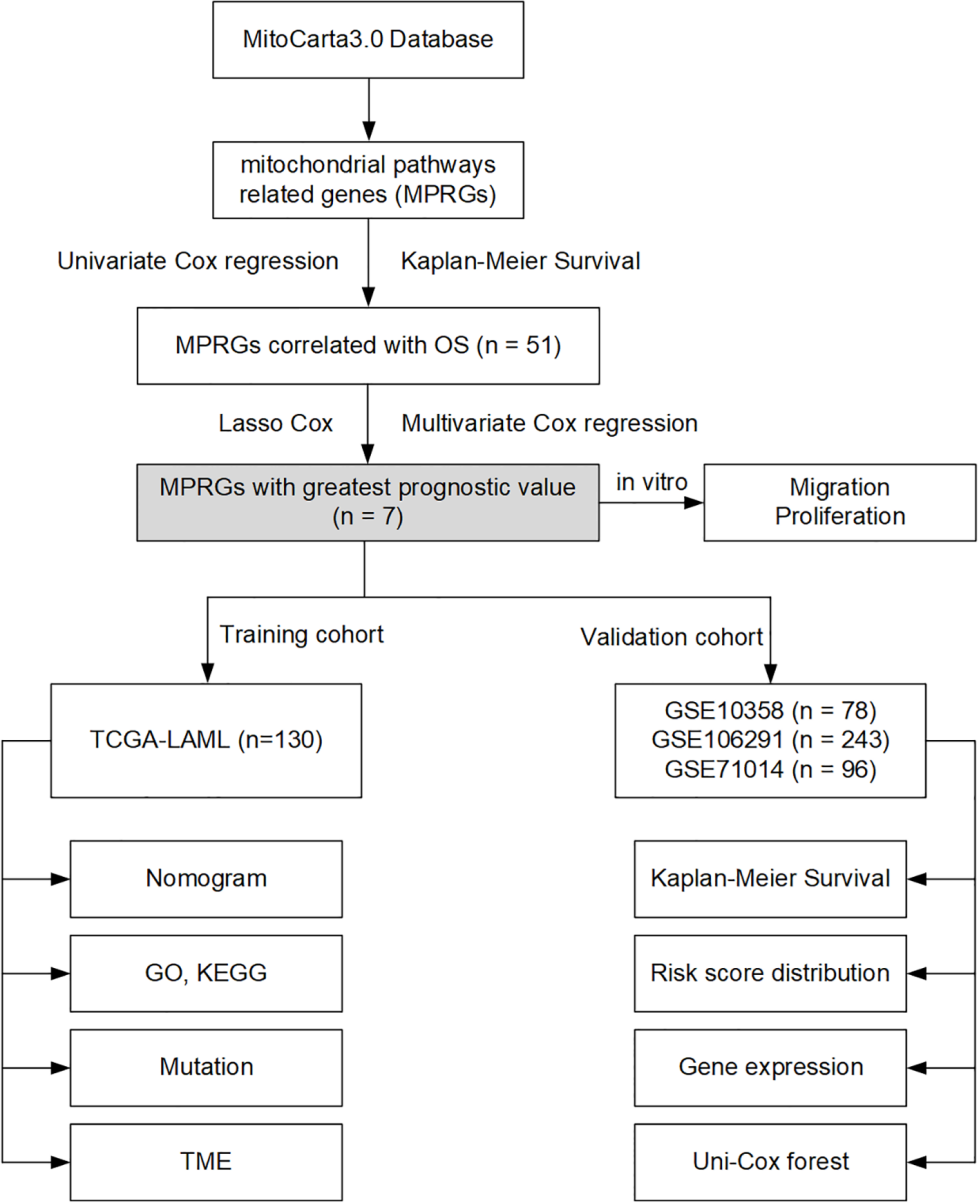
The general workflow of the current study. Mitochondria pathway related genes (MPRGs) were downloaded from the MitoCarta3.0 database. Univariate Cox regression and Kaplan-Meier survival analyses were applied to select MPRGs with prognostic potential. Finally, the risk model was constructed by multivariate Cox regression using genes obtained after LASSO regression. The risk model was validated in several datasets, and the function of UCP2 was confirmed by *in vitro* cellular experiments. OS, overall survival; LASSO, least absolute shrinkage, and selection operator; TME, tumor microenvironment.

**Figure 1** outlines our analytical pipeline. Mitochondria pathway related genes (MPRGs) were downloaded from MitoCarta3.0. Univariate Cox regression and Kaplan-Meier survival analyses were applied to select MPRGs with prognostic potential. LASSO regularization refined candidate selection, culminating in multivariate Cox model assembly. External cohort validation and UCP2 functional characterization completed the workflow.

### 3.2 Prognostic potential analysis of MPRGs

**Figure 2:**
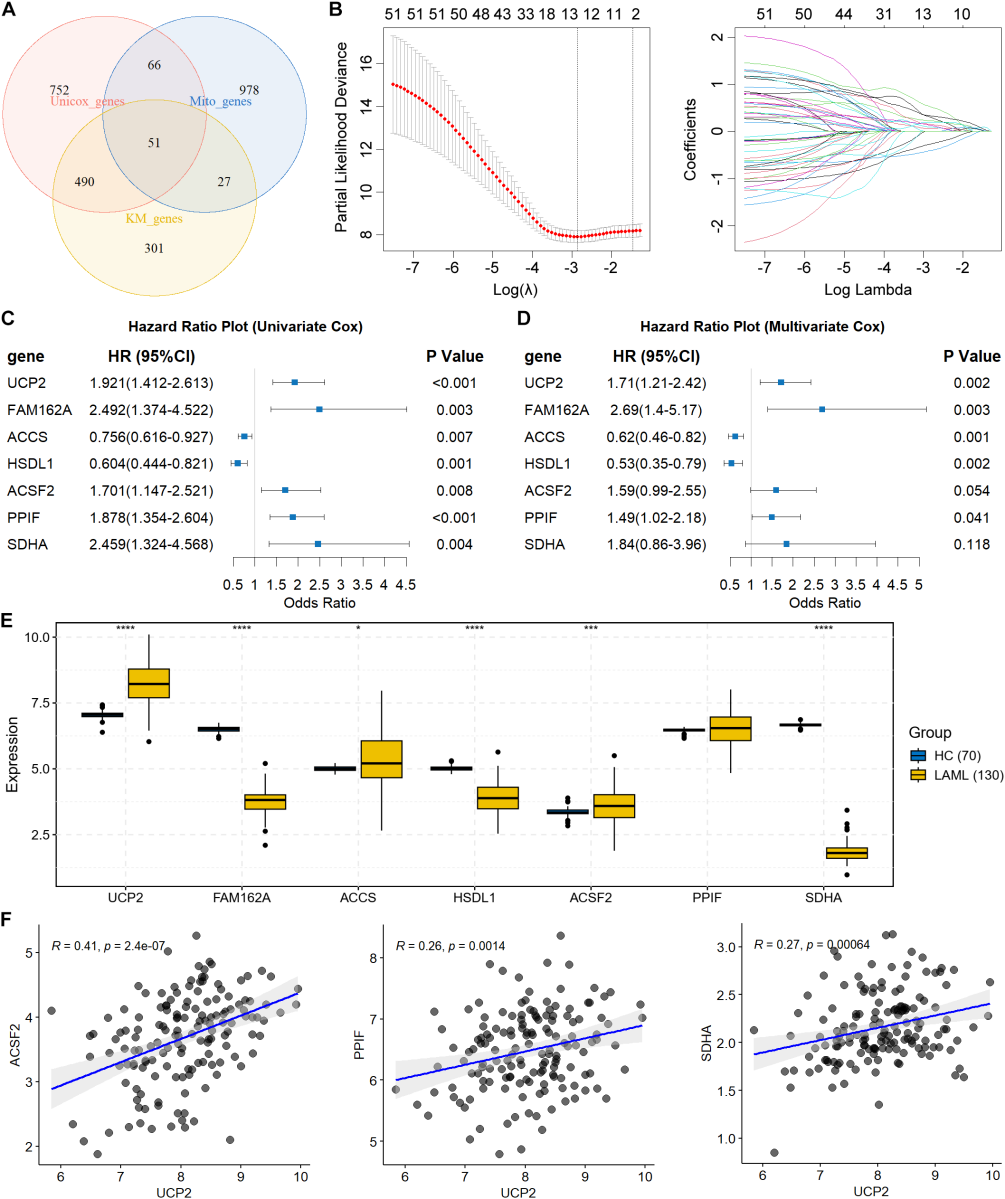
Identification of MPRGs signature in AML. **(A)** Intersection among MPRGs with prognostic values in both Univariate Cox regression and Kaplan-Meier survival analysis. **(B)** The LASSO coefficient profiles generated by 51 prognostic MPRGs. **(C, D)** Univariate and multivariate Cox regression analysis of the seven MPRGs with OS in AML patients from TCGA. **(E)** Expression of the seven MPRGs in AML samples and normal control samples. **(F)** Correlation between UCP2 with ACSF2, PPIF, and SDHA. *P < 0.05, ***P < 0.001, ****P < 0.0001.

Survival association screening via tinyarray revealed 1,359 MPRGs reaching significance (*P* < 0.01) in univariate Cox analysis (**Supplementary Table 2**) and 869 MPRGs in Kaplan-Meier testing (**Supplementary Table 3**). The intersection yielded 51 candidates demonstrating prognostic relevance by both criteria (**Figure 2A**).

### 3.3 Identification of MPRGs signature in AML

Sequential LASSO penalization and stepwise selection identified optimal predictors (**Figure 2B**). The finalized seven-gene signature comprised UCP2 (Uncoupling Protein 2), ACCS (1-aminocyclopropane-1-carboxylate synthase homolog), FAM162A (Family with sequence similarity 162 member A), HSDL1 (hydroxysteroid dehydrogenase like 1), ACSF2 (Acyl-CoA synthetase family member 2), PPIF (peptidylprolyl isomerase F), SDHA (Succinate dehydrogenase complex flavoprotein subunit A). Cox modeling confirmed each gene’s independent association with survival (**Figures 2C, D**). Among these genes, UCP2, ACCS, ACSF2 were increased in AML compared with normal control samples, while FAM162A, HSDL1, and SDHA were decreased in AML (**Figure 2E**). Correlation analysis showed that UCP2 was significantly correlated with several other signature genes, including ACSF2, PPIF, and SDHA, suggesting partial coordination among these MPRGs in AML samples (**Figure 2F**).

### 3.4 Validation of MPRGs signature in AML

**Figure 3:**
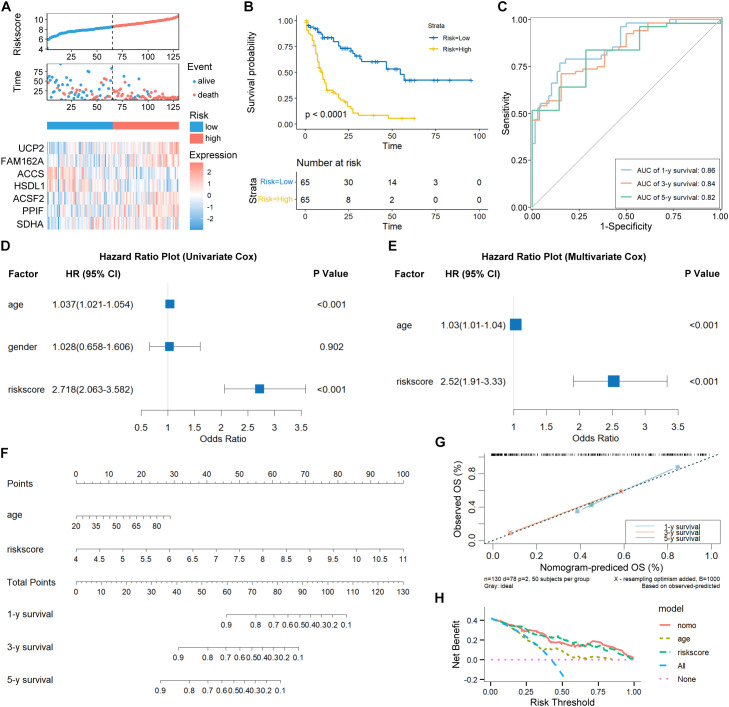
Validation of MPRGs signature in AML. **(A)** Risk score and patient distribution, and expression of risk genes in the TCGA-AML cohort. **(B)** Kaplan-Meier curve of the high- and low-risk patients. **(C)** The ROC curve for 1-, 3- and 5-year survival in TCGA-AML patients. **(D, E)** Univariate **(D)** and multivariate **(E)** Cox regression analysis of the risk score and clinical factors for AML patients. **(F)** The nomogram plot was constructed based on clinical factors and the risk score. **(G)** Calibration plot of the nomogram in TCGA-AML patients. **(H)** DCA curve of the nomogram, age, and risk score for the TCGA-AML patients.

Risk scores followed the formula: Riskscore = 0.5368048*UCP2 + 0.9896156*FAM162A - 0.4849365*ACCS - 0.6420198*HSDL1 + 0.4639932*ACSF2 + 0.3981706*PPIF + 0.6113426*SDHA. TCGA-LAML patients stratified by median score showed characteristic expression patterns: UCP2, FAM162A, ACSF2, PPIF, and SDHA elevated in high-risk cases while ACCS and HSDL1 reduction (**Figure 3A**). Survival curves confirmed significantly abbreviated outcomes for high-risk patients (**Figure 3B**). Time-dependent ROC yielded AUCs of 0.86, 0.84, and 0.82 at 1-, 3-, and 5-year endpoints (**Figure 3C**).

Multivariable modeling established the risk score as an independent survival determinant alongside age (**Figures 3D, E**). A composite nomogram facilitated individualized survival probability estimation at 1-, 3-, and 5-year intervals (**Figure 3F**), with calibration curves and DCA confirming predictive reliability and clinical utility (**Figures 3G, H**).

**Figure 4:**
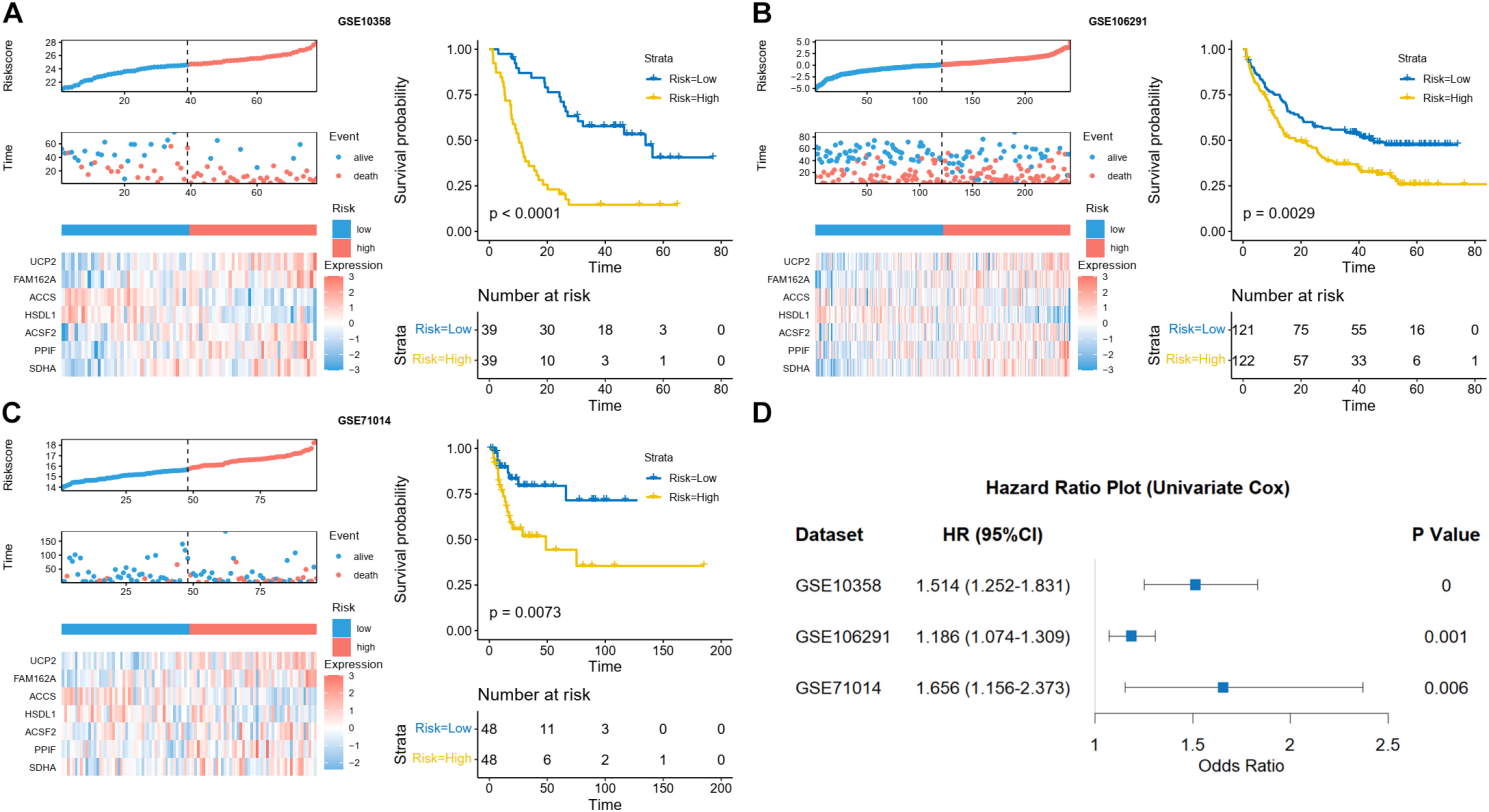
External validation of MPRGs signature in AML. Kaplan-Meier curve of the high- and low-risk patients, risk score and patient distribution, and expression of risk genes in GSE10358 **(A)**, GSE106291 **(B)**, and GSE71014 **(C)**. **(D)** Forest plot of univariate Cox regression analysis of the risk score AML patients in all three validation cohorts.

External validation in GSE10358, GSE106291, and GSE71014 recapitulated these findings: high-risk designation portended inferior survival, and gene expression patterns paralleled TCGA observations (**Figures 4A–C**). Univariate Cox analysis verified risk score significance across all validation cohorts (**Figure 4D**). The baseline clinical characteristics of the TCGA-LAML cohort and the three external validation cohorts are presented in **Supplementary Table 1**.

### 3.5 Functional enrichment and mutational landscape analysis

**Figure 5:**
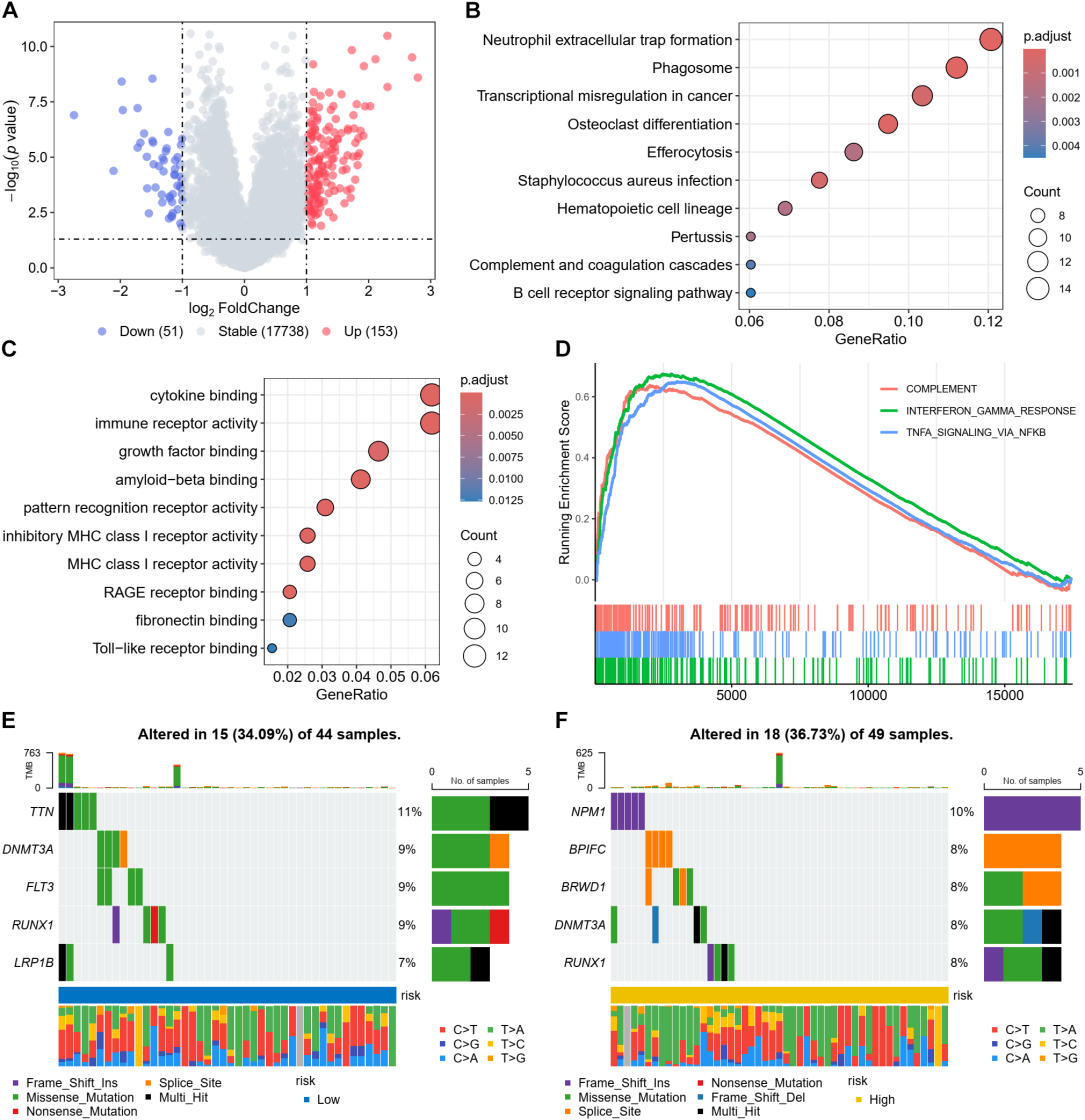
Functional enrichment and mutational landscape analysis. **(A)** Volcano plot displaying DEGs between high- and low-risk groups. Red dots represent significantly upregulated genes, and blue dots represent significantly downregulated genes in the high-risk group. The thresholds were set at |log2 fold change| > 1 and p < 0.05. KEGG **(B)**, GO **(C)**, and GSEA **(D)** analyses of DEGs. **(E, F)** Oncoplot showing the mutational landscape and top mutated genes in the low-risk group **(E)** and high-risk group **(F)**.

To explore the biological differences, we identified differentially expressed genes (DEGs) between high- and low-risk patients. A total of 204 DEGs were identified, comprising 153 upregulated and 51 downregulated genes in the high-risk group compared with the low-risk group (**Figure 5A**; **Supplementary Table 4**). KEGG pathway enrichment analysis revealed that these DEGs were significantly enriched in neutrophil extracellular trap formation, phagosome, hematopoietic cell lineage, and B-cell receptor cascades (**Figure 5B**). GO terms enriched immune-relevant functions: cytokine binding, immunoreceptor activity, MHC-I engagement, and Toll-like receptor interactions (**Figure 5C**). GSEA implicated complement activation, interferon-γ responsiveness, and TNF-α/NF-κB signaling (**Figure 5D**).

Mutational profiling revealed somatic alterations in 34.09% (low-risk) versus 36.73% (high-risk) of cases. Predominant mutations differed: TTN, DNMT3A, and FLT3 characterized low-risk patients, while NPM1, BPIFC, and BRWD1 prevailed in high-risk cases (**Figures 5E, F**).

### 3.6 Tumor microenvironment analysis

**Figure 6:**
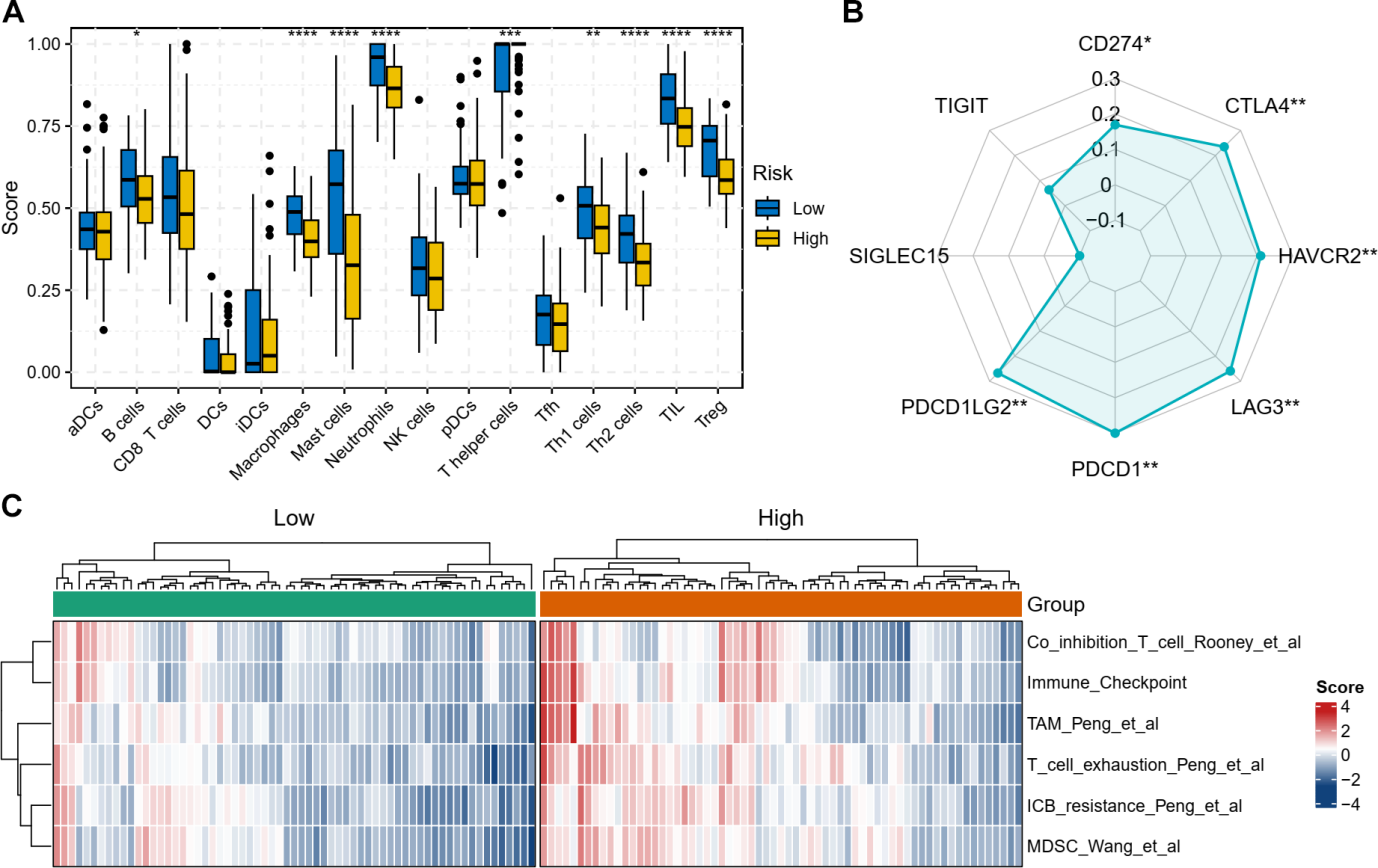
Tumor microenvironment analysis. **(A)** Comparison of immune cell infiltration levels between high-risk and low-risk patients. **(B)** Correlation analysis between the risk score and the expression of key immune checkpoint genes. **(C)** Enrichment scores of selected immune-related gene signatures in high-risk versus low-risk groups. TIL, tumor-infiltrating lymphocytes; Treg, regulatory T cells; MDSCs, myeloid-derived suppressor cells; TAMs, tumor-associated macrophages; ICB, immune checkpoint blockade. *P < 0.05, **P < 0.01, ***P < 0.001, ****P < 0.0001.

Immune contexture assessment using established signatures (19) suggested lower inferred abundance of B lymphocytes, macrophages, mast cells, neutrophils, helper T subsets (Th1, Th2), tumor-infiltrating lymphocytes, and regulatory T cells in high-risk patients (**Figure 6A**). Risk scores positively correlated with checkpoint molecules PDCD1, PDCD1LG2, LAG3, and CTLA4, though not SIGLEC15 or TIGIT (**Figure 6B**). Further gene set enrichment analysis with IOBR R package revealed that the high-risk group demonstrated elevated immunosuppressive signatures in high-risk cases: checkpoint pathway activation, MDSC accumulation, T-cell co-inhibition, exhaustion phenotypes, ICB resistance, and TAM enrichment (**Figure 6C**). These transcriptome-based findings suggest that high-risk patients may be associated with a more immunosuppressive immune microenvironment.

### 3.7 Targeting UCP2 inhibits AML cell growth and motility

**Figure 7:**
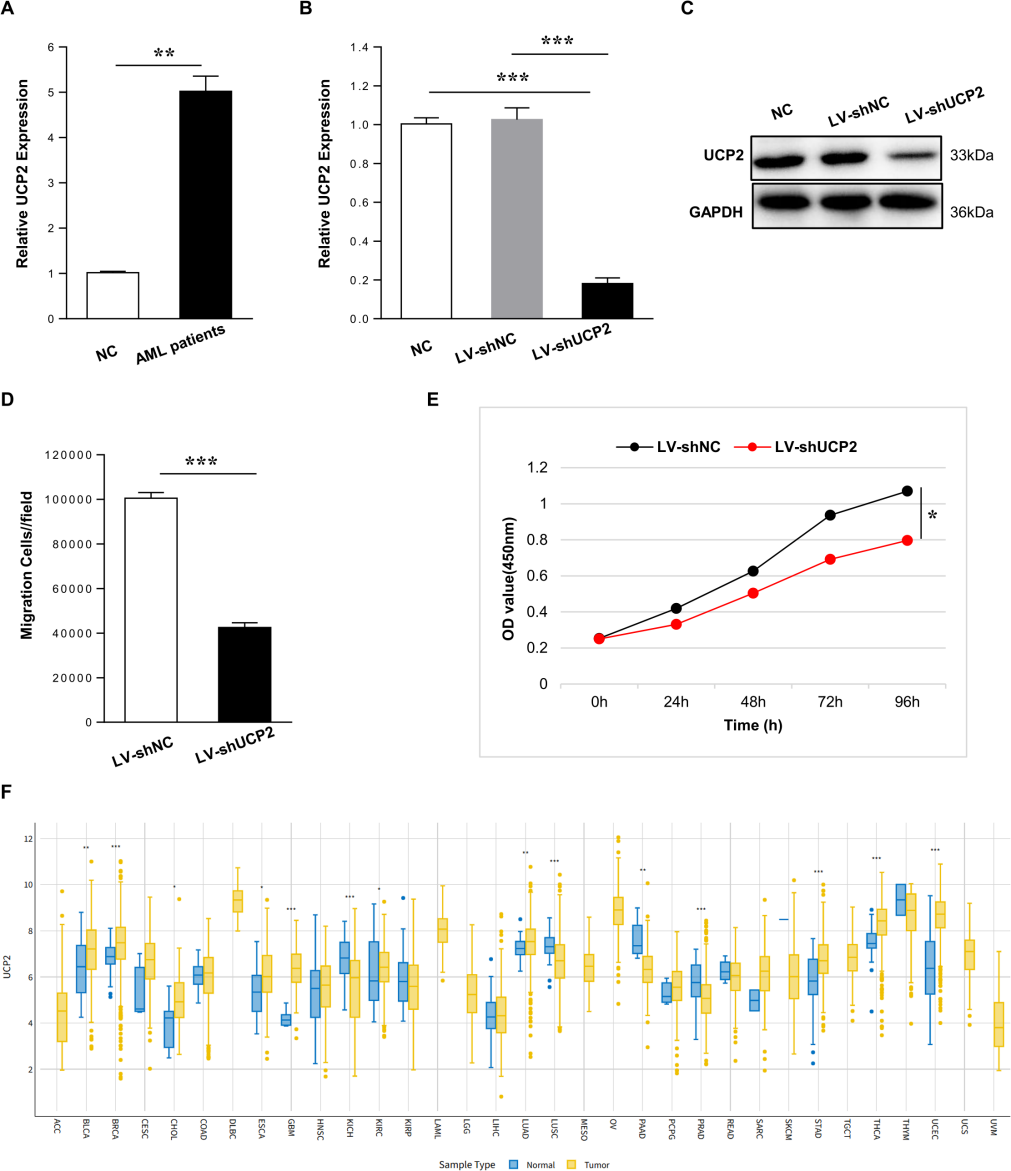
Targeting UCP2 inhibits AML cell growth and motility. **(A)** Validation of UCP2 overexpression in AML using qRT-PCR (n =10). **(B, C)** Efficient knockdown of UCP2 in MOLM-13 AML cells. **(B)** Quantification of UCP2 mRNA expression by qRT-PCR. **(C)** Representative western blot showing UCP2 protein levels in cells transduced with a non-targeting control shRNA (LV-shNC) or UCP2-targeting shRNA Lenti-virus (LV-shUCP2). **(D, E)** Functional consequences of UCP2 knockdown. **(D)** Cell migration was assessed by transwell assay. **(E)** Cell proliferation was measured by CCK-8. **(F)** Pan-cancer tumor-normal expression landscape of UCP2. *P < 0.05, **P < 0.01, ***P < 0.001.

UCP2 upregulation in AML was supported by preliminary validation in a small clinical cohort (**Figure 7A**). To assess its functional importance, we performed loss-of-function studies. Efficient knockdown of UCP2 using specific shRNA Lenti-virus was confirmed in MOLM-13 cells (**Figures 7B, C**). Functional assays demonstrated that UCP2 depletion markedly impaired both migratory and proliferative capacities (**Figures 7D, E**). These results provide preliminary loss-of-function evidence that UCP2 may contribute to AML cell proliferation and migration. To provide broader expression context, we further examined UCP2 expression across TCGA pan-cancer cohorts with available normal controls. This analysis showed that UCP2 is dysregulated in multiple tumor types, indicating that its upregulation is not unique to AML (**Figure 7F**).

## 4 Discussion

AML continues to pose a significant clinical challenge, largely due to its molecular heterogeneity and poor long-term survival outcomes (21, 22). Although our understanding of AML pathogenesis and treatment has advanced, progress in precise prognostic stratification and the identification of novel therapeutic targets has remained limited (23). In this study, we developed and validated a seven-gene prognostic signature based on mitochondrial pathway-related genes, which demonstrated robust predictive performance across multiple independent cohorts. Our results underscore the critical role of mitochondrial biology in AML prognosis and its close association with the tumor immune microenvironment. Several previous studies have reported mitochondria- or metabolism-related prognostic signatures in AML. Therefore, the present study should be viewed as an incremental integrative analysis rather than as a fundamentally new modeling framework.

The MPRG signature consists of seven genes: UCP2, FAM162A, ACCS, HSDL1, ACSF2, PPIF, and SDHA. Each gene contributes uniquely to mitochondrial metabolism and cellular homeostasis. UCP2, a mitochondrial inner membrane protein, decouples oxidative phosphorylation from ATP synthesis, thereby modulating reactive oxygen species (ROS) production and cellular metabolic activity (24). Previous studies have linked UCP2 to several malignancies, where it supports tumor cell survival by mitigating oxidative stress and enhancing metabolic adaptability (25). Downregulation of UCP2 played anti-cancer role in Breast cancer cells by Epicatechin (26). UCP2 influenced chemoresistance of Ovarian cancer via modulating the ROS dynamics (27). Our clinical and experimental data showed that UCP2 is upregulated in AML, and that its knockdown reduces MOLM-13 cell proliferation and migration. This effect may be related to altered mitochondrial redox balance, ROS production, or metabolic adaptability, although these possibilities were not directly tested in the present study and require further validation.

However, this finding should be interpreted with caution. Because our signature was derived from mitochondria pathway-related gene expression, it may reflect both mitochondrial abundance and gene-specific regulation. We therefore cannot determine whether the prognostic association of UCP2 is independent of mitochondrial content. Our pan-cancer tumor-versus-normal analysis also showed that UCP2 dysregulation is not unique to AML. Thus, UCP2 should be regarded as a biologically relevant component of the mitochondrial program in AML rather than an AML-specific marker. Further validation is needed.

SDHA is a core component of both TCA cycle and respiratory chain, directly connecting cellular metabolism to respiratory function (28). PPIF regulates mitochondrial permeability transition and is critically involved in modulating cell death pathways (29). Lactylation of the K182 site in ACSF2 results in mitochondrial dysfunction (30). Neutrophils expressing PPIF contribute to colorectal cancer progression via NETosis induced by mitochondrial ROS (31). The collective dysregulation of these genes likely functions synergistically, thereby promoting the metabolic reprogramming and enhancing the survival adaptability that are distinctive features of AML cells.

Our integrated analysis reveals a coherent immunosuppressive phenotype in high-risk patients. While high-risk tumors exhibit reduced infiltration of multiple immune cell types, including B cells, macrophages, mast cells, and T helper cells, they paradoxically display elevated expression of immune checkpoint genes such as PDCD1, PDCD1LG2, LAG3, and CTLA4. This observation suggests that although immune cells are sparse in the high-risk tumor microenvironment, the infiltrating lymphocytes exhibit exhaustion signatures. Importantly, high-risk patients demonstrated significantly elevated enrichment scores for MDSCs, T-cell exhaustion, ICB resistance, and TAM signatures, which may mechanistically explain both the sparse infiltration and T-cell dysfunction. MDSCs and TAMs actively inhibit lymphocyte recruitment, drive T-cell exhaustion, and confer resistance to immunotherapy (32, 33). Thus, high-risk patients represent an actively suppressed, myeloid-dominated microenvironment that may be refractory to conventional immunotherapeutic approaches.

The observed enrichment of immune-related pathways, such as neutrophil extracellular trap formation, complement activation, and interferon-γ response, in differentially expressed genes between high- and low-risk groups further substantiates the close relationship between mitochondrial dysfunction and immune dysregulation. Prior studies indicate that mitochondrial ROS and metabolites can regulate innate immune signaling and shape the polarization of tumor-associated macrophages (34). Thus, our results imply that targeting mitochondrial pathways could exert dual effects: directly impairing AML cell survival and simultaneously reprogramming the immunosuppressive tumor microenvironment.

The mutational landscape differed markedly between risk groups. Low-risk cases harbored predominant TTN, DNMT3A, and FLT3 alterations, whereas high-risk patients showed NPM1, BPIFC, and BRWD1 mutation enrichment. Notably, FLT3 mutations were more frequent in the low-risk group, whereas NPM1 mutations were more common in the high-risk group. This pattern differs from the usual clinical impression and should be interpreted cautiously. Because our model was built from mitochondria pathway-related gene expression, it may capture biological features distinct from conventional mutation-based risk stratification. In addition, the prognostic impact of NPM1 and FLT3 alterations may vary with co-mutation patterns and clinical context. While BPIFC and BRWD1 remain less studied in AML, their enrichment in high-risk patients indicates potential involvement in leukemogenesis or metabolic adaptation, warranting further investigation (35). Overall, the distinct mutational profiles observed between MPRG-based risk groups indicate that mitochondrial pathway-related signatures may reflect a biological dimension not fully captured by conventional genetic markers alone.

Our study has several limitations. First, this was a retrospective analysis based mainly on public datasets, and standardized ELN risk stratification information was not consistently available. Therefore, prospective cohorts with complete cytogenetic, molecular, and ELN annotations are needed to determine whether the MPRG signature provides prognostic value beyond established clinical risk systems. Second, immune infiltration was inferred from transcriptomic signatures and was not validated by flow cytometry or single-cell analysis of AML bone marrow samples. Third, the experimental validation of UCP2 was preliminary and limited to one AML cell line and one knockdown strategy. Additional AML models, independent shRNAs, overexpression or rescue experiments, and mitochondrial functional assays will be needed to clarify the mechanism. Finally, the biological roles of the other six signature genes remain to be investigated.

## 5 Conclusion

In summary, we established a prognostic signature based on mitochondrial pathway-related genes that stratified AML patients into groups with different clinical outcomes. This signature was associated with immune microenvironment features, including T-cell exhaustion and myeloid-related immunosuppressive signatures. Functional experiments further provided preliminary evidence that UCP2 may contribute to AML cell proliferation and migration. These findings support the prognostic relevance of mitochondrial pathway-related genes in AML and provide a basis for future studies investigating their biological and therapeutic implications.

## Data Availability

The original contributions presented in the study are included in the article/**Supplementary Material**. Further inquiries can be directed to the corresponding author/s.
